# Vanadium-Containing Anionic Chelate for Spectrophotometric Determination of Hydroxyzine Hydrochloride in Pharmaceuticals

**DOI:** 10.3390/molecules28062484

**Published:** 2023-03-08

**Authors:** Gergana Kirilova Kirova, Zdravka Yancheva Velkova, Vassil Borisov Delchev, Kiril Blazhev Gavazov

**Affiliations:** 1 Department of Chemical Sciences, Faculty of Pharmacy, Medical University of Plovdiv, 120 Buxton Bros Str., 4004 Plovdiv, Bulgaria; 2 Faculty of Chemistry, University of Plovdiv ‘Paisii Hilendarskii’, 24 Tsar Assen St., 4000 Plovdiv, Bulgaria

**Keywords:** hydroxyzine, vanadium(V), 6-hexyl-4-(2-thiazolylazo)resorcinol, spectrophotometric determination, liquid–liquid extraction, ion association, DFT calculations, pharmaceutical dosage forms

## Abstract

Four azo dyes known to form anionic complexes with V(V) were investigated as potential liquid–liquid extraction–spectrophotometric reagents for the antihistamine medication hydroxyzine hydrochloride (HZH). A stable ion-association complex suitable for analytical purposes was obtained with 6-hexyl-4-(2-thiazolylazo)resorcinol (HTAR). The molar absorption coefficient, limit of detection, linear working range, and relative standard deviation in the analysis of real pharmaceutical samples (tablets and syrup) were 3.50 × 10^4^ L mol^−1^ cm^−1^, 0.13 μg mL^−1^, 0.43–12.2 μg mL^−1^, and ≤2.7%, respectively. After elucidating the molar ratio in the extracted ion-association complex (HZH:V = 1:1), the ground-state equilibrium geometries of the two constituent ions—HZH^+^ and [VO_2_(HTAR)]^−^—were optimized at the B3LYP level of theory using 6-311++G** basis functions. The cation and anion were then paired in four different ways to find the most likely structure of the extracted species. In the lowest-energy structure, the VO_2_ group interacts predominantly with the heterochain of the cation. A hydrogen bond is present (V–O···H–O; 1.714 Å) involving the terminal oxygen of this chain.

## 1. Introduction

**Figure 1:**
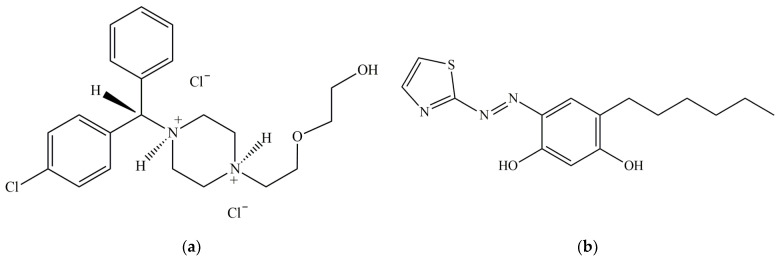
Molecular structure of hydroxyzine dihydrochloride (HZH) [2,4,26] (**a**) and 6-hexyl-4-(2-thiazolylazo)resorcinol (HTAR) [24,25] (**b**).

Hydroxyzine hydrochloride (HZH; IUPAC name: 2-[2-[4-[(4-chlorophenyl)-phenylmethyl]piperazin-1-yl]ethoxy]ethanol dihydrochloride; Figure 1a) is a first-generation antihistamine medication (histamine H_1_ blocker) that interferes with the binding of histamine in capillaries, as well as in bronchial and gastrointestinal smooth muscles. It has antiallergic, antispasmodic, antiemetic, sedative, and anxiolytic properties [1,2,3] and has been widely used in recent years [4], especially in the treatment of tension, anxiety, bronchial asthma, and allergic skin reactions.

Like any drug, HZH should be used judiciously. Drowsiness, dizziness, headache, dry mouth, constipation, and skin rash are common side effects associated with its use. Combinations with alcohol, lithium, and other sedatives, or accidental overdose, can cause hallucinations [5,6]. Because of HZH’s potential to cause more severe specific adverse effects, it is on the NCQA’s list of high-risk medications for the elderly [7].

Analytical methods of varying complexity and cost have been applied to determine HZH. Common examples include thin-layer chromatography [8], high-performance liquid chromatography [9], gas chromatography [10], capillary zone electrophoresis [11], acid–base titration [12], conductometric titration [13], differential pulse anodic voltammetry [14], and spectrophotometry [15,16,17,18,19,20,21].

The principal advantages of UV–vis spectrophotometry are its simplicity, flexibility, universality, and availability in terms of equipment, personnel, and consumables. Table 1 lists recent spectrophotometric procedures for the determination of HZH. The most sensitive one is based on a Cr(III) complex dissolved in acetone [16]. However, it is quite tedious and time-consuming, requiring drying in a vacuum dryer for 6 h.

Here, we report the development of a simple, fast, sensitive, and robust liquid–liquid extraction–spectrophotometric procedure for the determination of HZH in pharmaceuticals. It is based on an anionic complex of 6-hexyl-4-(2-thiazolylazo)resorcinol (HTAR; Figure 1b) with V(V). HTAR is a novel reagent for V(V), Cu(II), and Co(II) [22,23,24]. Like other azo derivatives of resorcinol, it can form an anionic chelate with V(V) in slightly acidic media, [VO_2_(HTAR^2−^)]^−^, which tends to form chloroform-extractable ion-association complexes with appropriate cations [25].

HZH is a colorless, water-soluble salt that can form mono- or dications, depending on the acidity. Information is available in the literature on both the crystal structure [4] and ionization constants (p*K*_a1_ = 1.93 and p*K*_a2_ = 7.52) [26]. It is a “spherical-type ion association reagent” according to Tôei’s classification [27], which exists as a monocation (HZH^+^) in slightly acidic media.

A novelty of the present work is the use of a vanadium complex for the determination of an active cationic component in real pharmaceutical samples. The selected chromophore ligand (HTAR) has a specific action that distinguishes it from other similar ligands in terms of the possibility of forming a stable and extractable ternary ion-association complex with the target cation. The application of quantum chemistry methods to elucidate the structure of the extracted species is another important aspect of this study.

## 2. Results and Discussion

### 2.1. Choice of Azo Dye

Four azo derivatives of resorcinol (ADR) were studied as part of a liquid–liquid extraction system containing V(V), HZH, and chloroform: 4-(2-pyridylazo)resorcinol (PAR), 4-(2-thiazolylazo)resorcinol (TAR), 4-(2-thiazolylazo)orcinol (TAO), and HTAR. These ARDs are known to form anionic chelates with V(V) in slightly acidic media [25,28,29,30,31,32,33]. It was found in our preliminary experiments that only HTAR was able to form extractable species with V(V) and HZH. The most likely reason for this is the presence of a hydrophobic tail with an appropriate orientation in its molecule (Figure 1b). Therefore, all further studies were conducted with this ADR.

The good extractability of the V(V)–HTAR–HZH ternary complex could be attributed to the effective hydrophobic envelope around the central V(V) ion and other hydrophilic regions. The bulkiness and spatial shape of both the ligand (HTAR) and the cation (HZH^+^) are essential for this [27,34,35]. Apparently, only the V(V)–HTAR anionic complex possesses the needed structural features favoring its appropriate positioning relative to the cation.

### 2.2. Absorption Spectrum

**Figure 2:**
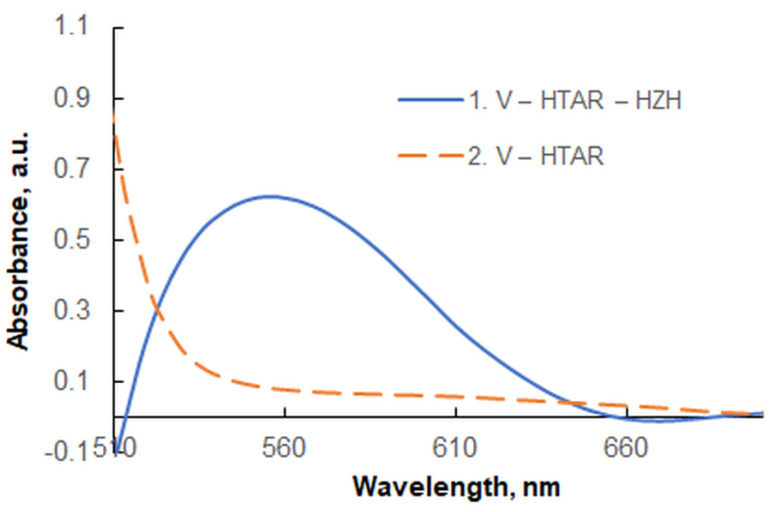
Absorption spectra in chloroform of the V(V)-HTAR-HZH complex (1) and the blank V(V)-HTAR (2); 3 × 10^−5^ mol L^−1^ of V(V), 2.4 × 10^−4^ mol L^−1^ of HTAR, 1.7 × 10^−5^ mol L^−1^ of HZH, pH of 4.3 (ammonium acetate buffer), extraction time of 45 s.

Figure 2 shows the absorption spectrum of the V(V)–HTAR–HZH complex in chloroform, measured against a blank. The absorption maximum is located at 554 nm. Its position coincides with that of a previously studied ion-association complex of [VO_2_(HTAR)]^−^ with a colorless cation derived from xylometazoline hydrochloride (XMZ) [25]. The spectral similarity indicates that the two complexes, V(V)–HTAR–HZH and V(V)–HTAR–XMZ, have the same ion-associative nature and contain the same chromophore anion: [VO_2_(HTAR)]^−^.

It is known that the color properties of the typical ion-association complexes represent a combination of the color properties of the constituent ions [35,36]. This can be used to simulate the visible spectra of extracted ion-association complexes containing colorless cations using theoretical data for the parent anions. Comparison of theoretical and experimental spectra of new complexes of this type is a convenient tool for clarifying their composition, structure, conformation, oxidation state of the central atom, etc. [37,38].

### 2.3. Effect of pH

**Figure 3:**
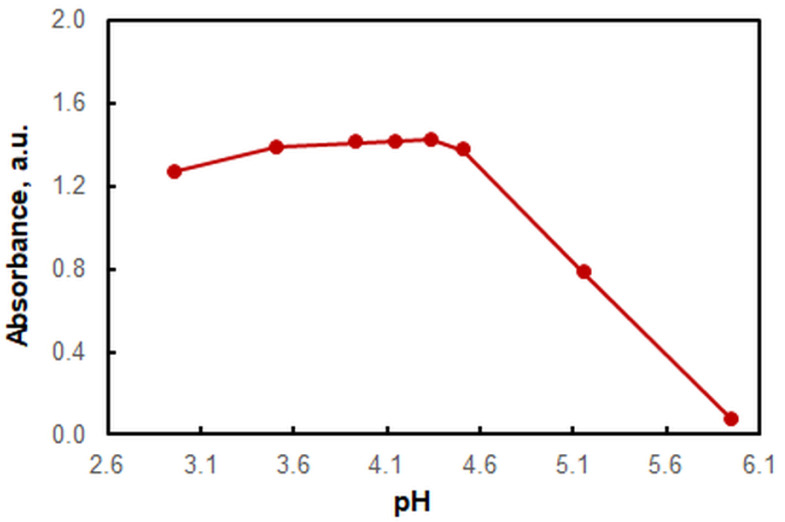
Effect of pH on the absorbance of the chloroform extract; 3 × 10^−5^ mol L^−1^ of V(V), 3 × 10^−4^ mol L^−1^ of HTAR, 1 × 10^−4^ mol L^−1^ of HZH, extraction time of 45 s, *λ* = 554 nm.

The effect of pH on the ion-association formation is shown in Figure 3. The experiments were carried out in the presence of ammonium acetate buffer. The analytical signal was maximal and constant in a relatively wide pH-interval: 3.5–4.5. The optimum pH value was around 4.3. This value falls within the range of maximum buffering capacity (pH 4.75 ± 1) [39] but is far enough from the breakpoint at a pH of 4.5.

### 2.4. Effects of Extraction Time

**Figure 4:**
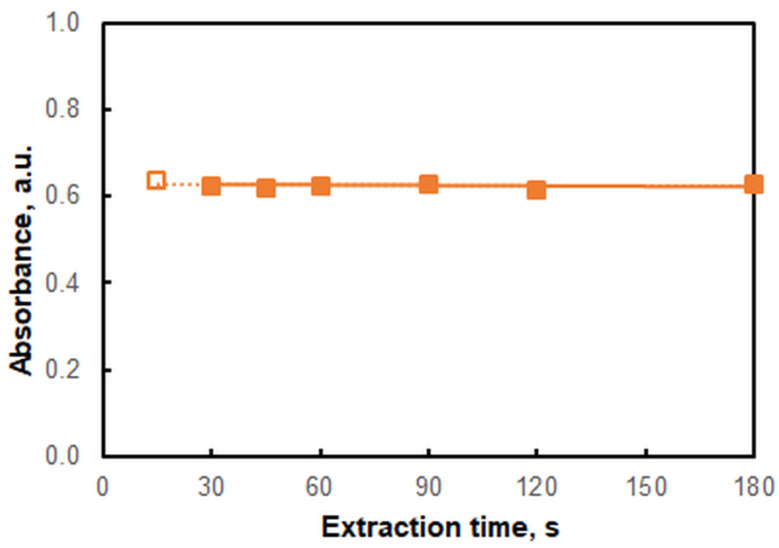
Effect of extraction time; 3 × 10^−5^ mol L^−1^ of V(V), 2.4 × 10^−4^ mol L^−1^ of HTAR, 1.7 × 10^−5^ mol L^−1^ of HZH, pH of 4.3 (ammonium acetate buffer), *λ* = 554 nm. The empty marker on the left indicates a slow phase separation process.

The effect of extraction time was studied in the range of 15 to 180 s (Figure 4). When the extraction time was short (e.g., 15 s) the phases separated slowly. A time of 30 s was sufficient to provide stable absorbance values. For greater reliability and ease of phase separation, we extended the extraction time to 45 s. This period is shorter than that required to extract the ternary V(V)–HTAR–XMZ complex (8 min) [25] and the methyl orange–HZH ion pair (3 min) [15]. 

### 2.5. Choice of V(V) and HTAR Concentrations

The concentration of V(V) in the aqueous phase was set to 3 × 10^−5^ mol L^−1^. At a 10-fold molar excess of HTAR, this concentration provided absorbances (at *λ*_max_ = 554 nm) of no more than 1.6 absorbance units. Three series of experiments were performed at three different HTAR concentrations: 2 × 10^−4^ mol L^−1^, 2.4 × 10^−4^ mol L^−1^, and 3 × 10^−4^ mol L^−1^. The selected optimal value was 2.4 × 10^−4^ mol L^−1^ (eightfold excess). This provided stable results and a wide linear range of the calibration plot (see Section 2.8).

**Figure 5:**
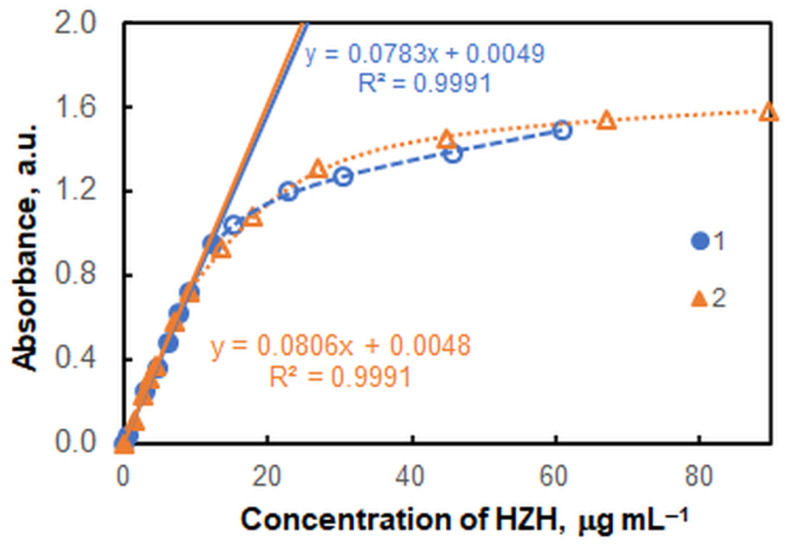
Saturation curves with HZH at two different HTAR concentrations: 2.4 × 10^−4^ mol L^−1^ (blue markers) and 3.0 × 10^−4^ mol L^−1^ (orange markers); 3 × 10^−5^ mol L^−1^ of V(V), pH of 4.3, extraction time of 45 s, λ = 554 nm. The full markers refer to the linear portions of the curves.

Figure 5 shows two of the obtained saturation curves. The slopes of the linear parts and the obtained correlation coefficients are practically the same. However, the linear range for the eightfold excess of HTAR over V(V) is wider.

### 2.6. HZH-to-V(V) Molar Ratio, and Formula of the Extracted Complex

**Figure 6:**
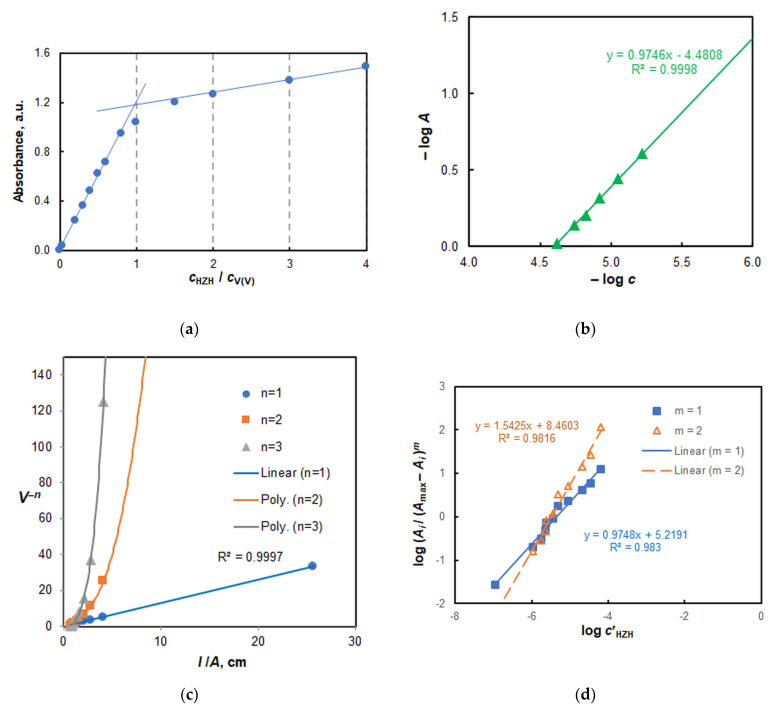
Determination of the HZH-V(V) molar ratio by the molar ratio method (**a**), Bent–French method (**b**), Asmus method (**c**), and mobile equilibrium method (**d**); 3 × 10^−5^ mol L^−1^ of V(V), 2 × 10^−4^ mol L^−1^ of HTAR, extraction time of 45 s, *λ* = 554 nm.

The HZH-to-V(V) molar ratio was determined at two HTAR concentrations: 2 × 10^−4^ mol L^−1^ and 3 × 10^−4^ mol L^−1^. The following four methods were used (Figure 6): the molar ratio method [40], the Bent–French method [41], the Asmus method [42], and the mobile equilibrium method [43]. The results strongly indicate that *n*(V):*n*(HZH) = 1:1. This ratio matches our prior expectations based on the view that at the optimal pH value V(V) should exist as [VO_2_(HTAR)]^−^ and HZH should exist as HZH^+^.

Figure 6d shows that the extracted ion-association complex does not dimerize—as do, for example, complexes obtained in systems containing V(V)–TAO [32,33]. Therefore, it can be represented by the formula (HZH^+^)[VO_2_(HTAR)].

### 2.7. Ground-State Equilibrium Geometries of the Ions, Calculated Energies, and Spectral Comparison

**Figure 7:**
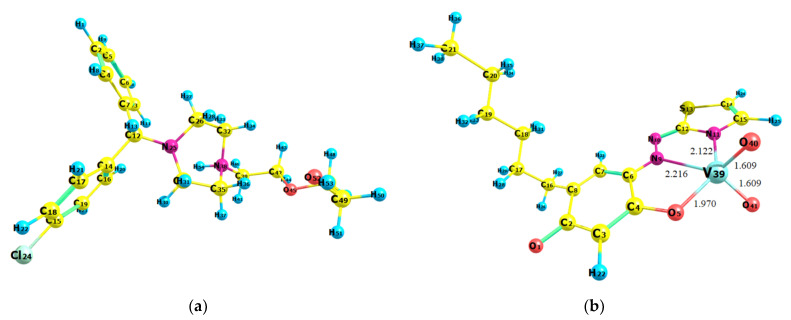
B3LYP-optimized ground-state equilibrium geometries of the cation (**a**) and anion (**b**). Visualization using the Chemcraft graphical program (https://chemcraftprog.com (accessed on 1 February 2023)).

**Figure 8:**
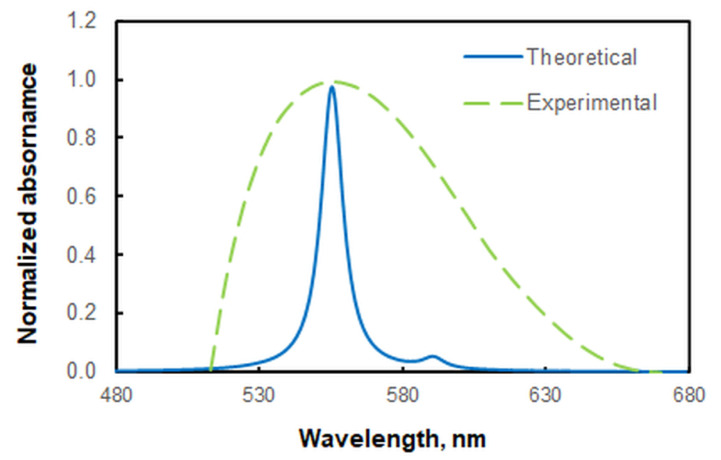
Comparison of the experimental spectrum of the extracted complex with a simulated spectrum (B3LYP/6-311++G** level of theory) of its anion moiety. A Lorentzian broadening and a scaling coefficient of 1.17 were used for the theoretical spectrum.

The ground-state equilibrium geometries of the two associating ions were found at the B3LYP/6-311++G** level of theory (Figure 7). The vertical excitation energies were then calculated for the anion [VO_2_(HTAR)]^−^ to simulate a theoretical UV–vis absorption spectrum. A good agreement of this spectrum with the experimental spectrum of the extracted ion-association complex was obtained, with a scaling coefficient of 1.17 (Figure 8). The theoretical absorption maxima in the visible region are shown in Table 2. The experimental band is mainly a result of π → π* electron transitions with different oscillator strengths *f* (0.5214 and 0.0200). The spectroscopically dark ^1^nπ* excited state has a rather low *f* value, suggesting its negligible influence. Another reason for the observed asymmetry of the experimental band is the stronger influence of the blank at shorter wavelengths (see Figure 2).

### 2.8. Optimized Ground-State Structures of the Ion-Association Complex

**Figure 9:**
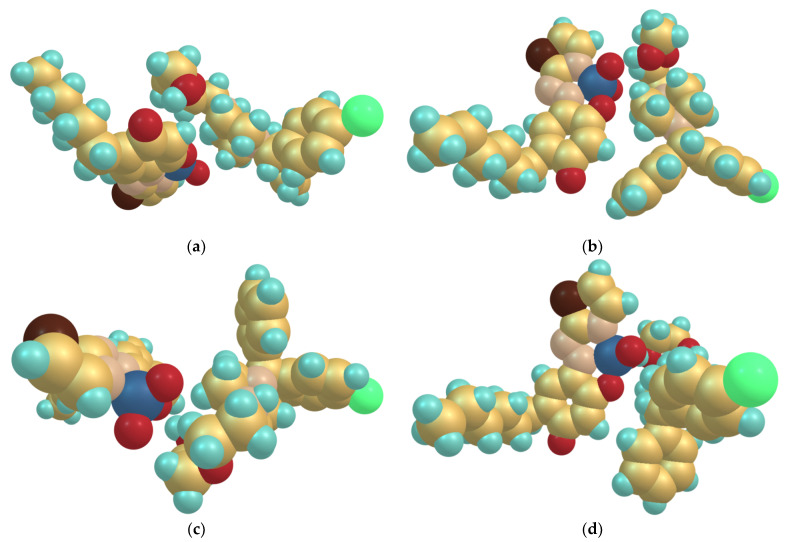
Optimized ground-state structures of the ion-association complex at the HF/6-31G level of theory: (**a**) Str. 1, *E*_1_ = –3909.6590 a.u.; (**b**) Str. 2, *E*_2_ = –3909.6660 a.u.; (**c**) Str. 3, *E*_3_ = –3909.6644 a.u.; (**d**) Str. 4, *E*_4_ = –3909.6633 a.u. Visualization using the Chemcraft graphical program (https://chemcraftprog.com (accessed on 1 February 2023)).

The next step was to pair the cation and anion properly. For this purpose, four different structures were constructed and fully optimized at the HF/6-31G level of theory (Figure 9). The calculated energies of these structures increased in the following sequence: *E*_2_ < *E*_3_ < *E*_4_ < *E*_1_. Therefore, the most likely structure of the ion-association complex is Str. 2 (Figure 9b). It is stabilized by a hydrogen bond between an oxygen of the VO_2_ group and the OH group of the cationic part.

**Figure 10:**
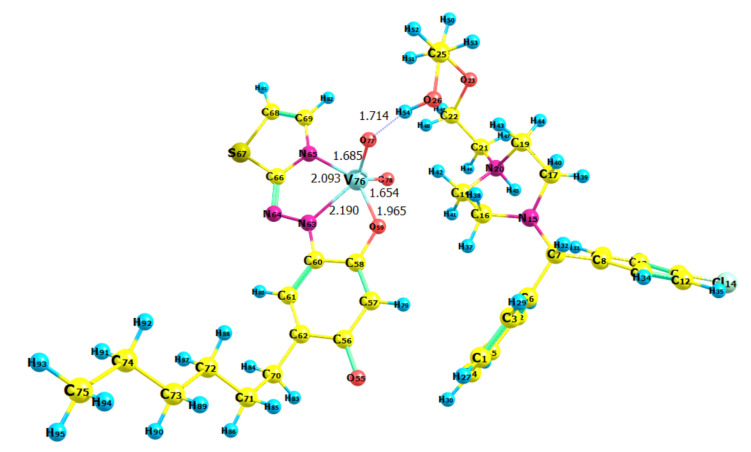
Reoptimized Str. 2 at the BLYP/6-31G level of theory. Visualization using the Chemcraft graphical program (https://chemcraftprog.com (accessed on 1 February 2023)).

Str. 2 was further reoptimized at a higher level of theory (BLYP/6-31G), and its structure is presented in Figure 10. The comparison between Str. 2 and that of the isolated chromophore anion (Figure 7b) shows that the ion association induces only minor changes in the bond lengths in the coordination entity. The calculated length of the hydrogen bond between the two associated ions (V76–O77···H54–O26) was 1.714 Å.

### 2.9. Chemical–Analytical Characteristics and Application

The correlation between the absorbance (*A*) and the HZH concentration was studied under the optimal conditions shown in Table 3. A very good linearity was observed for concentrations ranging from 0.43 to 12.2 μg mL^−1^ (*R*^2^ = 0.9991, *n* = 7; Figure 5). The linear regression equation was *A* = 0.0783*γ* + 0.005, where *γ* is the mass concentration in μg mL^−1^. The standard deviations of the slope and intercept were 0.0009 and 0.006, respectively. As can be seen, the intercept is statistically indistinguishable from zero. The limit of detection (LOD) and limit of quantitation (LOQ), calculated as 3 and 10 times the standard deviation of the blank (*n* = 10) divided by the slope, were 0.13 μg mL^−1^ and 0.43 μg mL^−1^, respectively. The values of the molar absorption coefficient and Sandell’s sensitivity were 3.50 × 10^4^ L mol^−1^ cm^−1^ and 12.8 ng cm^−2^, respectively. The logarithm of the extraction constant calculated by the mobile equilibrium method [38] (Figure 6d, curve 1) was log *K*_ex_ = 5.35 ± 0.17. Statistically indistinguishable results for log *K*_ex_ were obtained by two other methods: the Holme–Langmyhr method [44] (5.38 ± 0.10), and the Harvey–Manning method [45] (5.37 ± 0.18).

The method was tested for the determination of HZH in Neurolax^®^ tablets (Helax Healthcare Pvt. Ltd., Roorkee, India) with a claimed HZH content of 25 mg per tablet. The result was statistically indistinguishable from the above mentioned value: 25.3 ± 0.6 (mean ± SD; four replicate analyses). It was also identical to the result obtained by another method [15]: 25.2 ± 0.9 (mean ± SD, *n* = 4). To assess the interday reproducibility, eight replicate analyses of the same solution were additionally performed on the following two consecutive days (four analyses per day). The pooled results for the three days indicated that the reproducibility was satisfactory: 24.9 ± 0.8, RSD = 3.2%.

Furthermore, the scope of the studies was extended by the analysis of Neurolax^®^ tablets from another batch and of a syrup obtained by mixing the standard and other ingredients according to a known recipe [46,47]. The results obtained by these studies with unspiked and spiked samples are shown in Table 4.

The robustness of the method was evaluated by comparing the results obtained at different HTAR concentrations (see Figure 5). The equation of the calibration curve remained practically unchanged under these conditions. However, the dynamic interval was shorter when the HTAP concentration was 3 × 10^−4^ mol L^−1^; the limit of linearity (LOL) was 9.0 µg mL^−1^.

Another experiment related to the robustness assessment was a comparison of results at different extraction times: 45 s and 120 s. The results obtained were statistically identical.

## 3. Materials and Methods

### 3.1. Reagents and Chemicals

Reagents were purchased from Merck (Schnelldorf, Germany) and used without further purification as aqueous solutions. Standard solutions of HZH (*M*_r_ 447.83) were prepared at concentrations of 3 × 10^−4^ mol L^−1^ and 1 × 10^−4^ mol L^−1^. The ADRs (HTAR, PAR, TAR, and TAO) were dissolved in the presence of KOH [24,25]; *c*_ADR_ = 2 × 10^−3^ mol L^−1^. The V(V) solution (3 × 10^−4^ mol L^−1^) was prepared from NH_4_VO_3_ (puriss. p.a.). The pH of the aqueous phase was kept constant using a buffer solution prepared by mixing appropriate proportions of 2 mol L^−1^ solutions of ammonia and acetic acid. Distilled water and redistilled chloroform were used during the work.

### 3.2. Instrumentation

Ultrospec 3300 pro and Spectronic Camspec M550 UV–Vis scanning spectrophotometers (Garforth, United Kingdom), equipped with 1 cm cells, were used for the spectrophotometric measurements. The pH was checked using a WTW InoLab 7110 pH meter (Weilheim, Germany) with a glass electrode.

### 3.3. Samples

Neurolax^®^ tablets of two batches (A and B; both containing 25 mg of HZH per tablet as the active ingredient) were purchased from local pharmacies. Twenty tablets per batch were weighed and powdered with a mortar and pestle. Two test solutions (A and B) with an expected HZH concentration of 1 × 10^−4^ mol L^−1^ were prepared by adding water to a weighed portion of the powder and shaking for several minutes.

The syrup (50 mL, 2 mg mL^−1^ HZH) was prepared in our laboratory by mixing HZH, ethanol, sucrose, sodium benzoate, levomenthol, hazelnut flavor, and water [46,47]. An aliquot of this solution was further diluted with water to obtain the desired HZH concentration (1 × 10^−4^ mol L^−1^).

All solutions were stored in dark bottles at 4 °C and used within no more than 5 days.

### 3.4. Optimization Procedure

Solutions of V(V), ammonium acetate buffer, HTAR, and HZH were mixed in a 125-mL separatory funnel. The sample was diluted with water to a total volume of 10 mL. Then, a 4 mL portion of chloroform was added using a burette. The funnel was stoppered and shaken for a fixed time interval to transfer the complex to the chloroform phase. After the phase separation, a portion of the organic layer was poured into the cell. Absorbance was measured against chloroform or a simultaneously prepared blank containing no HZH.

### 3.5. Procedure for the Determination of HZH

Solutions of V(V) (1 mL, 3 × 10^−4^ mol L^−1^), buffer (1 mL, pH 4.3), and HTAR (1.2 mL, 2 × 10^−3^ mol L^−1^) were mixed in a 125 mL separatory funnel. An aliquot of the analyzed solution (containing 0.43–12.2 μg mL^−1^ of HZH) was added, and the sample was diluted with water until the total volume of the aqueous phase was 10 mL. Then, 4 mL of chloroform was added, and the funnel was shaken for 45 s. After phase separation, a portion of the organic layer was poured into the cell, and the absorbance was measured at 554 nm against a blank. The unknown HZH concentration was determined from a calibration plot prepared by the same procedure using standard solutions.

### 3.6. Theoretical

The ground-state equilibrium geometries of the ionic structures HZH^+^ and [VO_2_(HTAR)]^−^ were optimized at the B3LYP/6-311++G** level of theory. Subsequently, frequency calculations were performed to prove that the structures lay in minima. The vertical excitation energies of [VO_2_(HTAR)]^−^ were also calculated at this level. Furthermore, the cation and anion were paired in four different ways in neutral complexes distinguished by the mutual orientation of the constituents. Because of the large size of the systems, their stability was estimated by the computed energies at the HF/6-31G theoretical level. The most stable structure of the ion pair was finally reoptimized at the higher theoretical level—BLYP/6-31G. All calculations were carried out using the GAUSSIAN 16 program package [48]. The Chemcraft graphical program (https://chemcraftprog.com (accessed on 1 February 2023)) was used to visualize the results of the quantum chemistry computations [49].

## 4. Conclusions

A sensitive, precise, fast, and robust extraction–spectrophotometric method was developed for the determination of HZH. It was successfully applied to the analysis of pharmaceutical products. The method is based on an ion-association complex of the monocationic form of HZH and the anionic chelate of V(V) with a new commercially available azo dye—HTAR. Due to some peculiarities of its structure, the V(V)–HTAR anionic chelate exhibits selectivity for the extraction of HZH^+^, and the dye HTAR cannot be replaced by similar dyes, such as PAR, TAR, or TAO. Using quantum chemical calculations, the ground-state equilibrium geometry of the extracted species was optimized. A good match between the experimental spectrum and the simulated spectrum of the chromophore anion [VO_2_(HTAR)]^−^ is a guarantee of the correctness of the proposed structure.

## Figures and Tables

**Table 1:** Spectrophotometric procedures for the determination of HZH.

Reagent(s)	*λ*_max_, nm	Linear Range, μg mL^−1^	*ε*, L mol^−1^ cm^−1^	LOD	Comments	Ref.
Methyl orange	510	–	–	–	Includes both extraction and re-extraction. Chloroform consumption per sample: 20 mL	[15]
Reinecke salt	525.5	30–520	1.27 × 10^5^	–	A slow and tedious procedure involving precipitation and drying of a Cr(III) complex for 6 h	[16]
Diphenylcarbazone + Hg(II)	540	Up to 60	6.62 × 10^3^	–	An indirect determination based on the decrease in the absorption of the Hg(II)–diphenylcarbazone complex	[17]
Chloranilic acid	535	25–150	1.37 × 10^3^	1.25	A low-sensitivity procedure involving a charge-transfer complexation reaction in chloroform/acetonitrile	[18]
Orange II	480	1.5–15	2.07 × 10^4^	0.14	Based on a chloroform-extracted ion pair. Chloroform consumption per sample: 20 mL	[19]
Picric acid	400	3.75–45	8.06 × 10^3^	0.62	Based on a charge-transfer complexation reaction in chloroform	[20]
Iodine	380	1.25–15	2.59 × 10^4^	0.13	Based on a charge-transfer complexation reaction in dichloromethane. Short shelf life of the used reagent solution	[20]
–	230	–	–	–	Not used for the analysis of real samples	[21]
HTAR + V(V)	554	Up to 12.2	3.50 × 10^4^	0.13	A sensitive, fast, and robust extraction–spectrophotometric procedure based on a ternary ion-association complex	This work

**Table 2:** Vertical excitation energies and oscillator strengths of excited states of the anionic chelate calculated at the B3LYP/6-311++G** level of theory.

Excited State	Vertical Excitation Energy, nm	Oscillator Strength
^1^ππ*	504	0.0200
^1^ππ*	475	0.5214
^1^nπ*	452	0.0005

**Table 3:** Extraction–spectrophotometric optimization.

Optimized Parameter	Scope of Optimization	Optimal Value
Wavelength, nm	500–760	554
Concentration of V(V), mol L^−1^	–	3.0 × 10^−5^
Concentration of HTAR, mol L^−1^	(2–3) × 10^−4^	2.4 × 10^−4^
pH (ammonium acetate buffer)	3.0–6.0	4.3
Extraction time, s	15–180	45

**Table 4:** Determination (*n* = 4) of HZH in unspiked and spiked samples.

Sample	HZH Spike, μg mL^−1^	HZH Found, * μg mL^−1^	RSD, %	Recovery, %
Neurolax^®^ tablets (Batch 1)	0	3.04 ± 0.07	2.3	–
	1.5	4.58 ± 0.11	2.4	103
	3.0	6.07 ± 0.09	1.5	101
	4.5	7.52 ± 0.14	1.9	99.6
Syrup	0	2.99 ± 0.08	2.7	–
	1.5	4.44 ± 0.08	1.8	96.7
	3.0	6.03 ± 0.12	2.0	101
	4.5	7.46 ± 0.13	1.7	99.3

* mean ± SD.

## Data Availability

Not applicable.

## Sample Availability

Samples of the compounds are not available from the authors.
